# Suppression of *Sclerotinia sclerotiorum* by the Induction of Systemic Resistance and Regulation of Antioxidant Pathways in Tomato Using Fengycin Produced by *Bacillus amyloliquefaciens* FZB42

**DOI:** 10.3390/biom9100613

**Published:** 2019-10-16

**Authors:** Ayaz Farzand, Anam Moosa, Muhammad Zubair, Abdur Rashid Khan, Venance Colman Massawe, Hafiz Abdul Samad Tahir, Taha Majid Mahmood Sheikh, Muhammad Ayaz, Xuewen Gao

**Affiliations:** 1 College of Plant Protection, Department of Plant Pathology, Nanjing Agricultural University, Key Laboratory of Monitoring and Management of Crop Disease and Pest Insects, Ministry of Agriculture, Nanjing 210095, China; ayaz.farzand@uaf.edu.pk (A.F.); zubair_biotech@yahoo.com (M.Z.); malix.477@hotmail.com (A.R.K.); venmass2@yahoo.com (V.C.M.); hasamadtahir@gmail.com (H.A.S.T.); tahamajid1705@yahoo.com (T.M.M.S.); ayazbbt45@yahoo.com (M.A.); 2 Department of Plant Pathology, University of Agriculture, Faisalabad, P.O. Box 38040, Pakistan; annu_77@live.com

**Keywords:** lipopeptide, MALDI-TOF-MS, HPLC, reactive oxygen species, induced systemic resistance, defense-related genes

## Abstract

Lipopeptides from *Bacillus* species exhibit promising biological control activity against plant pathogens. This study aimed to explore the potential of purified fengycin to induce systemic resistance in tomato against *Sclerotinia sclerotiorum*. *Bacillus amyloliquefaciens* FZB42, its mutant AK1S, and their corresponding metabolites showed in vitro inhibition of *S. sclerotiorum* mycelium. Fengycin derived from an AK1S mutant was purified and identified through HPLC and MALDI-TOF-MS, respectively. Scanning electron microscopy (SEM) and transmission electron microscopy (TEM) revealed structural deformities in the fungal mycelium. Moreover, fengycin induced the accumulation of reactive oxygen species (ROS) in *S. sclerotiorum* mycelium and downregulated the expression of ROS-scavenging genes viz., superoxide dismutase (*SsSOD1*), peroxidase (*SsPO*), and catalase (*SsCAT1*) compared to the untreated control. Furthermore, the lesion size was dramatically reduced in fengycin-treated tomato plants compared to plants infected with *S. sclerotiorum* only in a greenhouse experiment. Additionally, the transcriptional regulation of defense-related genes *GST*, *SOD*, *PAL*, *HMGR*, and *MPK3* showed the highest upsurge in expression at 48 h post-inoculation (hpi). However, their expression was subsequently decreased at 96 hpi in fengycin + *S. sclerotiorum* treatment compared to the plants treated with fengycin only. Conversely, the expression of *PPO* increased in a linear manner up to 96 hpi.

## 1. Introduction

Biological control has emerged as the most effective, environment-friendly, and non-toxic option to control several plant pathogens [1,2]. *Bacillus* species are potential biological control agents against multiple soil-borne plant pathogens [3]. *Bacillus* species are considered as antimicrobial compound-manufacturing factories [4] with the potential to produce several broad-spectrum antifungal secondary metabolites [5,6]. *Bacillus amyloliquefaciens* FZB42 (now *B. amyloliquefaciens* subsp. *plantarum* FZB42) is a gram-positive bacterium well-recognized for its antagonistic potential, efficient rhizosphere colonization, and plant growth-promoting effect [7,8]. A genome analysis of *B. amyloliquefaciens* FZB42 has revealed 10 gene clusters, covering approximately 10% of the whole genome, that are responsible for manufacturing secondary metabolites with strong antimicrobial activity. Among these secondary metabolites, cyclic lipopeptides are the most significant. 

Lipopeptides are non-ribosomally-synthesized peptide derivatives [9,10] comprised of three main families or groups: iturin, surfactin, and fengycin [6]. Fengycin produced by *B. amyloliquefaciens* FZB42 has been reported to exhibit strong antifungal activity [11]. Fengycin primarily causes the suppression of filamentous fungi and inhibits phospholipase A2 [12]. Direct effects of fengycin include membrane disruption, the efflux of cellular contents, and ultimately the cell death of targeted microbes [13]. The amphiphilic nature and small size of the peptide portion of fungicide help during its insertion into the cell membrane and result in alteration of the membrane permeability. However, the accurate role of fengycin and intrinsic biochemical determinants involved in its antifungal activity have not been explicitly elucidated until now. 

Besides direct antagonism, *Bacillus* species protect plants against pathogens through the stimulation of induced systemic resistance (ISR). Lipopeptides produced by *Bacillus* have been reported to be involved in the stimulation of ISR in plants [14,15]. ISR is a systemic resistance response induced by rhizobacteria that can suppress pathogens and ultimately diseases in plants [16]. ISR induction triggers the production of plant defense-related proteins such as peroxidase (POD), lipoxygenase (LOX), chitinase (CHI), and β-1,3-glucanase (GLU) [17,18], and enzymatic antioxidants such as superoxide dismutase (SOD), phenylalanine lyase (PAL), and polyphenol oxidase (PPO) involved in plant defense [19,20,21]. Purified fengycin and surfactin can induce ISR in plants, as is evident from previous studies [22]. The importance of surfactin and fengycin in ISR induction in beans has been reported [23]. In another report, surfactin and fengycin elicited a defense response in ‘Valencia’ oranges against *Penicillium digitatum* and upregulated the expression of plant defense-related genes [24]. Surfactin and iturin induced ISR in plants and elicited the expression of defense-related proteins in strawberry against *Colletotrichum gloeosporioides*, suggesting the role of lipopeptides in plant defense activation [25]. Several other studies have also elucidated the role of lipopeptides in ISR elicitation [26,27,28]. Based on these reports, we can assume that fengycin could play a major role in plant defense elicitation. Therefore, to understand the role of fengycin in ISR elicitation and the transcriptional regulation of defense-related proteins, we have studied the expression *PAL*, *GST*, *SOD*, *HMGR*, and *MPK3* genes in tomato plants with or without treatment with fengycin. 

In order to develop biopesticides using antagonistic *Bacillus* strains, the mode of action of fengycin against fungal pathogens should be clearly understood. Several reports have presented the structure, synthesis, and antifungal activity of fengycin [5,29,30,31], but only a few reports have evaluated the antifungal effect of fengycin on fungal pathogens systematically. As an effort to understand the mechanism of action of fengycin, we were prompted to explore it’s antifungal and plant protective potential. The primary objective of the present study was to check whether fengycin could regulate defense-related genes and induce ISR in tomato plants against *Sclerotinia sclerotiorum* infection. In this study, we used fengycin purified and identified through HPLC and MALDI-TOF-MS, respectively. The results showed that besides exhibiting direct antifungal activity, fengycin produced by *B. amyloliquefaciens* FZB42 also induced oxidative burst in *S. sclerotiorum* mycelium. Additionally, it could also induce systemic resistance in tomato plants to protect them against this fatal pathogen. We hypothesize that the pathogen suppression and disease control in plants is the result of an orchestrated activity of fengycin to induce systemic resistance and upregulate the expression of several defense-related genes in tomato plants.

## 2. Materials and Methods 

### 2.1. Bacterial and Fungal Cultures

*B. amyloliquefaciens* (FZB42) wild type and its mutant AK1S used for the antifungal activity assay against *S. sclerotiorum* were obtained from the Biological Control and Bacterial Molecular Biology Lab, Nanjing Agricultural University, China, and stored at −80 °C in 60% glycerol solution. The bacterial strain and its mutant were streaked on Luria Bertani (LB) medium supplemented with 1.5% agar and incubated at 37 °C for further use. 

### 2.2. Antifungal Activity Assay

The antifungal activity of FZB42, AK1S mutant, and corresponding crude secondary metabolite extracts was evaluated in a dual culture assay. An *S. sclerotiorum* culture block (0.6 cm) was placed at the center of the Petri plates containing Potato Dextrose Agar (PDA) medium. Subsequently, 5 µL of an overnight culture of FZB42, AK1S, and their corresponding secondary metabolites was inoculated 3 cm away from the fungal culture block on a sterilized filter paper block. The plates were sealed with parafilm and incubated at 25 °C for 4 days. The experiment was repeated thrice, with three replicates for each treatment.

### 2.3. Extraction and Purification of Fengycin from B. Amyloliquefaciens FZB42 Mutant AK1S

AK1S mutant was inoculated on LB medium and incubated in a rotary shaker at 180 rpm for 24 h at 30 °C. Afterward, fermentation was carried out by inoculating Landy medium with 3 mL of AK1S mutant overnight culture and incubating it at 30 °C for 48 h in a rotary shaker at 180 rpm. The culture was centrifuged at 10,000× g at 4 °C for 30 min to obtain 100 mL cell-free supernatants. The supernatant was kept overnight at 4 °C after adjusting its pH to 2. Later, the precipitates were collected by centrifugation at 10,000× g at 4 °C for 30 min and dissolved in methanol [32]. Furthermore, these methanolic extracts were filtered through 0.2 µm filters. Fengycin was purified by using methanol and dichloromethane as solvent in a silica column in three different concentrations (*v*/*v*) (solution-1, 1:3 methanol/dichloromethane; solution-2, 3:1 methanol/dichloromethane; and solution-3, 5:1 methanol/dichloromethane). Finally, elutes obtained from solution-2 and solution-3 were analyzed with an HPLC-1200 system (Agilent Technologies, CA, USA) with a 5 µm column Agilent Eclipse XDB-C18. Acetonitrile with trifluoroacetic acid 0.1% (*v*/*v*) (Sigma-Aldrich, St. Louis, MO, USA) was mobile phase A, while Milli-Q water with triflouroacetic acid 0.1% (*v*/*v*) was mobile phase B. The flow rate of fengycin was maintained at 0.9 mL/min with a linear gradient of mobile phase A, developed over 60 min from 10% to 100%. The elution pattern of fengycin was determined by measuring the absorbance at 205 nm [33]. The elution components were determined by the matrix-assisted laser desorption/ionization-time of flight mass spectrometry (MALDI-TOF-MS) in a Bruker Daltonik Reflex MALDI-TOF-MS instrument for desorption and ionization with a 337 nm nitrogen laser [34]. The matrix was α-Cyano-4-hydroxycinnamic acid. 

### 2.4. Ultrastructural Changes in the Fungal Mycelium

Ultrastructural changes in the hyphae were observed through a scanning electron microscope (SEM) (Hitachi S-3000N, Tokyo, Japan) and transmission electron microscope (TEM) (Hitachi H-600, Tokyo, Japan). *S. sclerotiorum* hyphae were incubated for 18 h following treatment with 20 μg/mL of fengycin. Post incubation, *S. sclerotiorum* hyphae were fixed with 2.5% glutaraldehyde solution. Fungal hyphae were rinsed for 10 min thrice with 100 mM phosphate buffer after fixation. Fixed hyphae were post-fixed for 3 h in osmium tetroxide (1%), followed by dehydration using ethanol gradient. The samples were analyzed under a scanning electron microscope (Hitachi S-3000N, Tokyo, Japan) following a gold coating. For TEM analysis, an ultra-microtome was used to fix fungal hyphae in the section (Epon 812). The samples were analyzed following fixation under the transmission electron microscope (Hitachi H-600, Tokyo, Japan).

### 2.5. Assessment of Reactive Oxygen Species

The accumulation of reactive oxygen species (ROS) in *S. sclerotiorum* hyphae after treatment with fengycin was assessed by probe dichloro-dihydro-fluorescein diacetate (DCFH-DA) and fluorescence microscopy (Thermo Fisher Scientific, Hanover Park, IL, USA). Hyphae were treated with 20 μg/mL fengycin for 12 h and the methanol served as the control. Then, they were centrifuged at 1200× g for 8 min and subsequently resuspended in 10 mM sodium phosphate buffer (pH 7.4). The samples were incubated with 10 μM DCFH-DA for 30 min at 25 °C. Later, the samples were observed under a microscope (Olympus IX71, Tokyo, Japan) (excitation 488 nm, emission 535 nm). The experiment was repeated thrice, with three replicates under the same conditions.

### 2.6. Induction of ISR in Tomato Plants by Fengycin

In planta, an experiment was designed to check if purified fengycin extracted from AK1S can induce resistance against *S. sclerotiorum* in tomato plants. The experiment was comprised of four treatments: (1) healthy control (treated with 5% methanol solution *v*/*v*); (2) *S. sclerotiorum* only (infected control); (3) fengycin only; and (4) fengycin + *S. sclerotiorum*. Tomato seeds were surface-sterilized with 2% NaOCl and placed in a sterilized Petri plate containing wet filter papers. For seed germination, the plates were incubated in a 8 h light and 16 h dark period for 5 days at 25 °C. Post-germination, the roots of tomato seedlings (in treatment with fengycin and fengycin + *S. sclerotiorum*) were dipped in 5% methanolic solution containing 100 µg/mL of fengycin. The roots of infected control plants (*S. sclerotiorum* only) and healthy control plants were dipped in 5% methanol before transplantation. Four weeks post-transplantation, the roots of these plants were treated again with fengycin (100 µg/mL) in 5% methanolic solution by root drenching one day prior to inoculation with the pathogen. The roots of plants in the healthy control and *S. sclerotiorum* only were treated with 5% methanol. Later, the stems of tomato plants were inoculated by wounding the plant at the center of the stem with a sterilized toothpick. Furthermore, a 6 mm block of the fungal culture with a very thin layer of PDA medium was excised and placed over this wound and sealed with a parafilm. The stem of the plants in fengycin-only treatment was wounded and inoculated with a thin layer of PDA medium and sealed with parafilm. The experiment was conducted thrice, with five replicates of each treatment. Disease progress was assessed by measuring the size (cm) of lesions on the stem 4 days post-pathogen inoculation.

### 2.7. Extraction of Total RNA and Expression Analysis by RT-qPCR

To study the expression of ROS-scavenging genes (Table 1), total RNA was extracted from *S. sclerotiorum* mycelia after treatment with 20 µg/mL fengycin and methanol (in the case of control treatment) for 12 h by using RNAiso Reagent (Takara Biotechnology Co., Dalian, China), following the manufacturer’s guideline.

The expression of genes (Table 2) involved in tomato plant defense against *S. sclerotiorum* was studied by collecting leaf samples after 0, 48, and 96 h post-inoculation (hpi). Total RNA was extracted by using the plant RNA extraction kit (Omega Bio-tek, Norcross, GA, USA), according to the manufacturer’s protocol. The purity and concentration of RNA were measured by using NanoDrop 1000 (Thermo Scientific, Wilmington, DE, USA). First-strand cDNA was synthesized using Evo M-MLV with the gDNA clean RT kit (Accurate Biology, Hunan, China), according to the manufacturer’s manual. The expression of genes was studied in a Real-time Thermocycler (QuantStudio-6 Thermo Fisher Scientific, San Jose, CA, USA) using the SYBR Green Premix Taq HS qPCR kit (Accurate Biology, Hunan, China), following the producer’s instructions. The sequences of ROS genes were obtained from (https://genome.jgi.doe.gov/Sclsc1/Sclsc1.home.html) and defense genes in tomato were obtained from National Center for Biotechnology Information (NCBI) (https://www.ncbi.nlm.nih.gov). Actin was used as an internal control or housekeeping gene.

### 2.8. Statistical Analysis

All experiments were conducted in a completely randomized design. Experimental data was subjected to statistical analysis using SPSS statistical software. Means were separated using Tukey’s HSD test at *p* ≤ 0.05 after ANOVA.

## 3. Results

### 3.1. Antifungal Activity Assay

**Figure 1:**
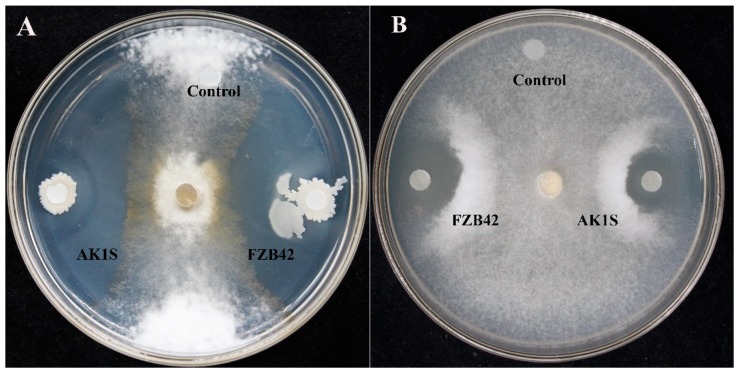
Antifungal activity of bacterial cultures and their crude extracts against *S. sclerotiorum* on Potato Dextrose Agar (PDA) medium: (**A**) direct antifungal activity of *Bacillus amyloliquefaciens* FZB42 wild type and AK1S mutant, and (**B**) antifungal activity of crude extracts from FZB42 and AK1S mutant.

The FZB42 wild type, AK1S mutant, and their corresponding secondary metabolite crude extracts showed antifungal activity against *S. sclerotiorum* by forming a clear inhibition zone around the fungal colony (Figure 1). The AK1S mutant, having the ability to produce fengycin only, was used to indicate the antifungal effect of fengycin against *S. sclerotiorum*.

### 3.2. HPLC- and MALDI-TOF-MS-Based Detection of Fengycin

**Figure 2:**
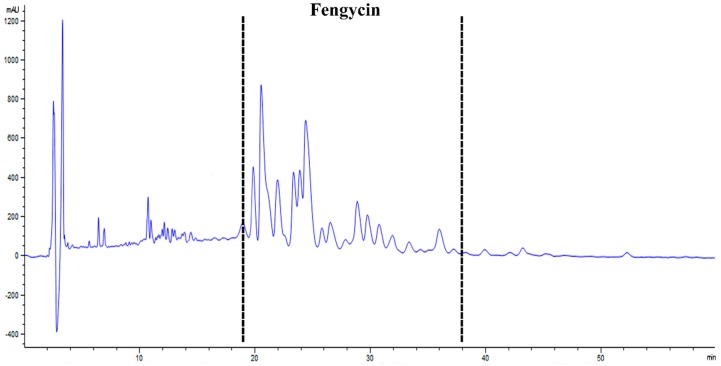
HPLC chromatogram of fengycin extracted and purified from AK1S mutant.

**Figure 3:**
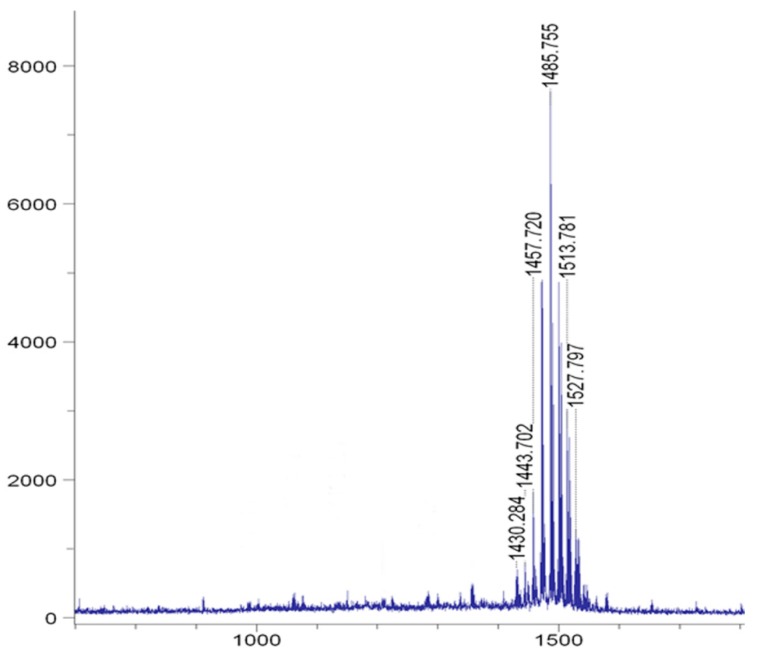
MALDI-TOF mass spectra of purified fengycin from AK1S mutant. C14, C16, and C17 homologues of fengycin were detected. The peaks in a range of masses from 1400 to 1600 *m*/*z* represent fengycin.

Fengycin was purified from AK1S mutant (which is a double mutant producing fengycin only and unable to produce surfactin and bacillomycin D). HPLC analysis of elutes collected from solution-3 showed peaks for fengycin between 20 and 37 min (Figure 2). Moreover, MALDI-TOF-MS analysis of the peaks confirmed the presence of fengycin and its homologs as the *m*/*z* ratio of the observed peaks was related to previously reported peaks of fengycin in the literature [11] (Figure 3). There were molecular ion peaks for C14 fengycin at *m*/*z* 1457.7(M + Na)^+^, C16 fengycin 1485.7 (M + Na)^+^, C16 fengycin 1513.8 (M + Na)^+^, and C17 fengycin 1527 (M + Na)^+^.

### 3.3. Fengycin Induced Ultrastructural Changes in S. sclerotiorum Hyphae

**Figure 4:**
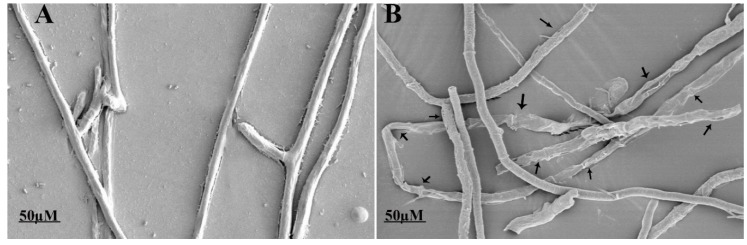
Effect of fengycin on the morphology of *S. sclerotiorum* under a scanning electron microscope: (**A**) control and (**B**) fengycin-treated (20 µg/mL) sample.

**Figure 5:**
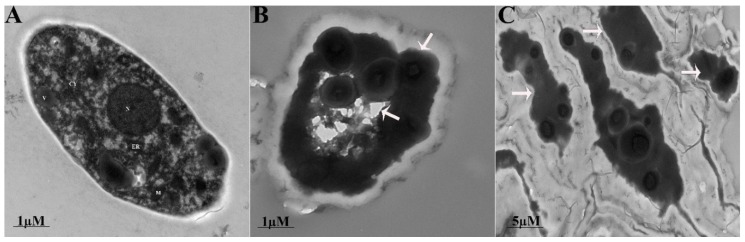
Ultra-structural deformities observed in the mycelium of *S. sclerotiorum* under a transmission electron microscope: (**A**) control (**B**,**C**) treated with fengycin (20 µg/mL). CY = cytoplasm, N = nucleus, ER = endoplasmic reticulum, V = vacuole, and M = mitochondria.

The ultrastructural changes in the morphology of fungal hyphae caused by fengycin were observed under SEM and TEM. The untreated hyphae of *S. sclerotiorum* appeared long, dense, and cylindrical in shape under SEM. Conversely, fungal hyphae treated with fengycin showed deformities or abnormalities in their hyphal morphology, including the curling, shrinkage, plasmolysis, pore formation, distortion, and breakdown of fungal hyphae (Figure 4). The results indicated the leakage of cellular contents as a result of fengycin treatment. TEM analysis was performed to confirm the ultrastructural changes in the cellular morphology of the pathogen. These results confirmed that the fungal hyphae treated with fengycin showed a loss of cellular integrity, cell shrinking, damage of the cell membrane, uneven thickness of the cell, displacement of cellular contents, and leakage of cytoplasmic material due to the breakdown of the cell wall and cell membrane (Figure 5). In contrast, untreated fungal hyphae had a well-defined cell wall, intact cell membrane, and septum. Moreover, they had clearly visible, uniformly distributed, and electro-dense cytoplasmic material. 

### 3.4. ROS Accumulation in S. sclerotiorum Hyphae

**Figure 6:**
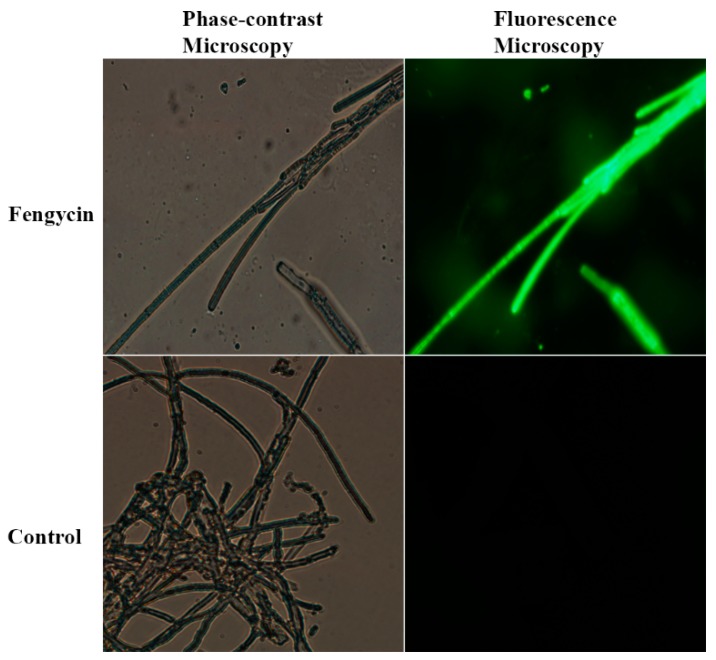
Effect of fengycin (20 µg/mL) on the production of ROS in *S. sclerotiorum* hyphae. Green fluorescence indicates ROS accumulation in *S. sclerotiorum* hyphae.

The effect of purified fengycin on the accumulation of ROS in fungal hyphae was assessed by using a DCFH-DA kit for ROS detection in fungal mycelium (Figure 6). Stronger green fluorescence was observed in the hyphae treated with fengycin compared to the untreated control. The green fluorescence was not only restricted to a few hyphae, but also expanded throughout the fengycin-treated mycelial network compared to the control treatment.

### 3.5. Expression of ROS-Scavenging Genes in S. sclerotiorum

**Figure 7:**
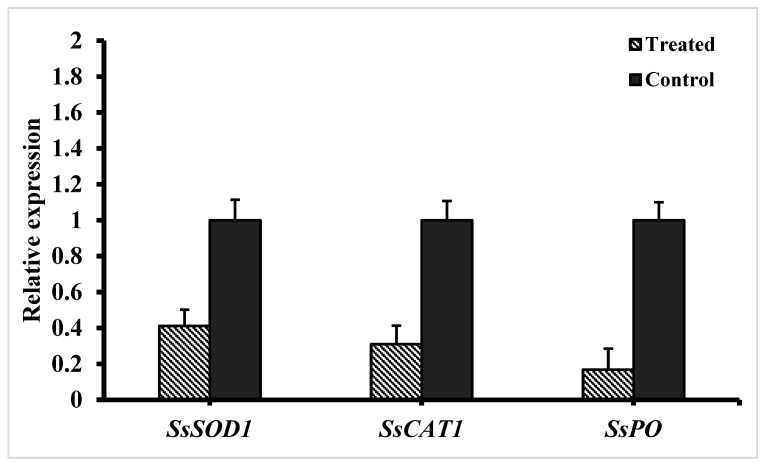
Expression analysis of ROS-scavenging genes in *S. sclerotiorum* mycelium after treatment with fengycin for 5 h. Mean values were analyzed and separated by Tukey’s HSD test at *p* ≤ 0.05 after one-way ANOVA. Error bars represent standard error ± SE of the mean.

The relative expression of ROS-scavenging genes in *S. sclerotiorum* was studied after treating the mycelium with fengycin for 12 h (Figure 7). The results revealed that the expression of ROS-scavenging genes superoxide dismutase (*SsSOD1*), peroxidase (*SsPO*), and catalase (*SsCAT1*) was significantly downregulated following treatment with fengycin. Moreover, among ROS-scavenging genes, peroxidase (*SsPO*) and catalase (*SsCAT1*) were the most downregulated genes.

### 3.6. Induction of ISR in Tomato Plants by Fengycin

**Figure 8:**
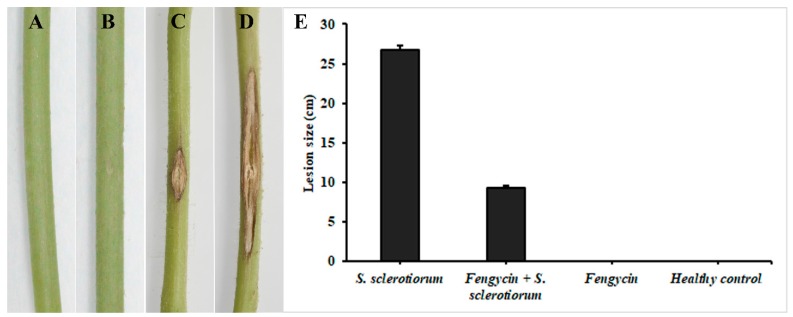
Fengycin-induced systemic resistance (ISR) in tomato plants against *S. sclerotiorum*. (**A**) healthy control, (**B**) fengycin only, (**C**) fengycin + *S. sclerotiorum*, (**D**) *S. sclerotiorum* only (infected control), and (**E**) lesion size (cm). Mean values were analyzed and separated by Tukey’s HSD test at *p* ≤ 0.05 after one-way ANOVA. Error bars represent standard error ± SE of the mean.

The effect of fengycin on ISR induction was examined by the plant’s response against *S. sclerotiorum* infection. The lesion development at the stem of tomato plants was measured after 4 days of inoculation. Plants infected with *S. sclerotiorum* only showed typical symptoms of infection, displaying the development of necrotic lesions on the stem (Figure 8). The results revealed a statistically significant difference in lesion size following treatment with fengycin compared to *S. sclerotiorum*-infected plants at *p* ≤ 0.05. Plants infected with *S. sclerotiorum* and treated with fengycin showed significantly less development of the necrotic lesion area compared to plants infected with *S. sclerotiorum* only.

### 3.7. RT-qPCR Analysis of Defense-Related Genes in Tomato

**Figure 9:**
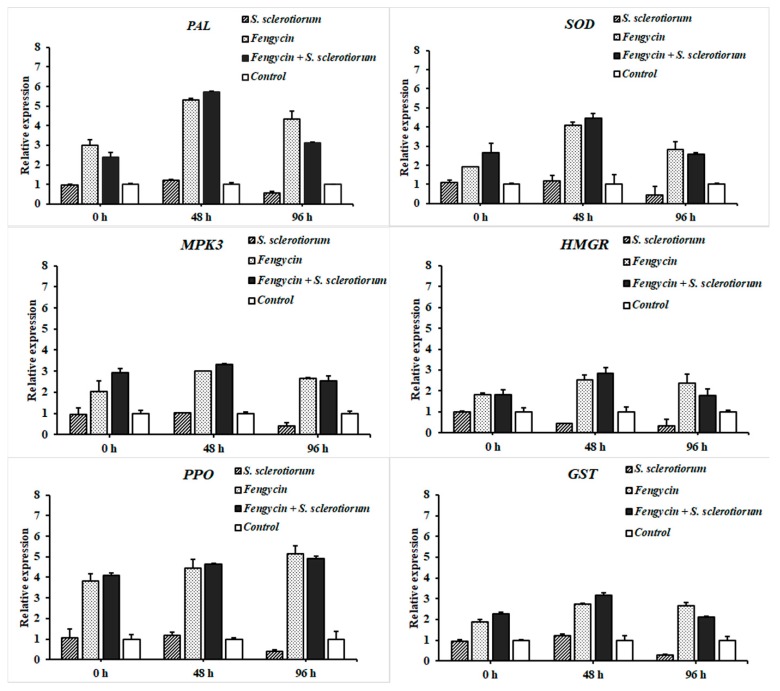
Expression profiling of six defense-related genes in tomato plants at 0, 48, and 96 h post-inoculation. Mean values were analyzed and separated by Tukey’s HSD test at *p* ≤ 0.05 after one-way ANOVA. Error bars represent standard error ± SE of the mean.

The expression of defense-related genes was studied in tomato plants through RT-qPCR analysis at 0, 48, and 96 hpi. The results revealed the differential expression of genes involved in the defense activation of tomato plants in a time frame analysis (Figure 9). The highest expression of genes, including *PAL*, *GST*, *SOD*, *HMGR*, and *MPK3*, was observed at 48 hpi in plants treated with fengycin. Interestingly, tomato plants treated with fengycin and challenged with the pathogen showed maximum upregulation of these genes after 48 hpi compared to the plants treated with fengycin only. However, the expression of these genes was downregulated at 96 hpi in fengycin + *S. sclerotiorum* treatment compared to the plants treated with fengycin only. Moreover, unlike other genes, *PPO* showed a varied pattern as its expression kept increasing in a linear manner up to 96 hpi in fengycin-treated plants. Furthermore, the expression of all genes was significantly downregulated in plants infected with *S. sclerotiorum* only at 96 hpi compared to the healthy control. 

## 4. Discussion

*Bacillus* are beneficial bacteria that provide direct or indirect protection against several fungal pathogens. *Bacillus* rely on different mechanisms to antagonize the effect of plant pathogens. They produce several bioactive antifungal peptides involved in antibiosis, with promising potential for plant protection [35,36]. Antibiosis is the main mechanism of biological control that has been well-studied genetically and biochemically [35]. In recent years, several studies have reported the inhibition of fungal pathogens by cyclic lipopeptides [6,37,38]. The excretion of antimicrobial compounds by *Bacillus* species is the primary cause of the formation of fungal growth inhibition zones in dual culture [39]. Among antimicrobial compounds, lipopeptides are the key compounds from *Bacillus* species involved in the direct suppression of fungal pathogens [40]. Fengycin has already been described for its inhibitory effect on a range of phytopathogenic fungi, such as *Podosphaera fusca* [41], *Botrytis cinerea* [42], and *Fusarium graminearum* [11]. Therefore, in this study, we have attempted to reveal the mechanism of antifungal activity of fengycin against *S. sclerotiorum* and its involvement in defense elicitation in tomato plants.

Purified fengycin extracted from AK1S mutant (which is a double mutant and can produce fengycin D only and is unable to produce surfactin and bacillomycin D) was detected through HPLC and identified by MALDI-TOF-MS analysis. HPLC is widely used for the detection and high-quality purification of compounds in the fields of medicines and cosmetics [43]. HPLC and MALDI-TOF-MS have been used simultaneously for the extraction, purification, and detection of fengycin from *B. subtilis* CMB32 in an earlier report [44]. The detection of lipopeptides from whole-cell surface extracts of *Bacillus* species through MALDI-TOF-MS has been previously reported [45,46]. In this investigation, MALDI-TOF-MS confirmed the presence of C14, C16, and C17 homologues of fengycin produced by AK1S mutant. The range of masses belonging to fengycin observed in our study is similar to that of previous reports, where masses of *m*/*z* 1485 and 1527 [24], and 1513.78 were reported for fengycin [33]. Fengycin peaks that appear in a mass range of *m*/*z* 1400-1600 have been successfully detected by MALDI-TOF-MS in single protonated forms [43,47,48,49]. In support of our work, the detection of purified fengycin produced by AK1S mutant of *B. amyloliquefaciens* FZB42 through HPLC and its identification through MALDI-TOF-MS have been reported in a previous study from our lab [11]. 

SEM revealed ultrastructural changes in fungal hyphae viz., the curling, shrinkage, plasmolysis, pore formation, and breakdown of *S. sclerotiorum* hyphae due to fengycin produced by *B. amyloliquefaciens* FZB42. The results of SEM were further confirmed by TEM analysis. The present study is supported by previously [28] conducted research reporting that fengycin caused morphological changes in *Magnaporthe grisea*’s hyphal cell wall and cell membrane, as analyzed by SEM and TEM. Antifungal lipopeptides induce ultrastructural changes in plasma membranes of fungal hyphae, resulting in the leakage of cellular contents and cell death [50]. In another instance, the plasmolysis and shrinkage of *Lasiodiplodia theobromae* hyphae due to antifungal lipopeptides produced by *B. subtilis* B1 was reported, which is consistent with our findings [51]. Similarly, fengycin induced the deformation and breakage of *Fusarium graminearum* hyphae [11]. In several reports, fengycin has been reported to cause severe damage to the plasma membrane of hyphal cells, resulting in fungal cell death [52,53]. The possible mode of action for the damaging effects of fengycin on hyphal cells is the interaction of fengycin with sterol and phospholipid molecules of fungal plasma membranes that alters the membrane structure and permeability [54]. This explains the possible reason behind the adverse effects of fengycin on the hyphal structure of *S. sclerotiorum* in the present study.

Further study on the mechanism behind the control of pathogen by using fengycin indicated that fengycin induced a high accumulation of ROS in *S. sclerotiorum* hyphae. The accumulation of ROS in *S. sclerotiorum* mycelium was studied using a DCFH-DA kit for ROS detection following treatment with fengycin for 12 h. ROS at a low concentration work as intracellular messengers for several molecular events, while ROS at a high concentration can induce cell death due to ROS oxidative stress [55]. Similar to our findings, fengycin induced a burst of ROS coupled with cell death of *Magnaporthe grisea* hyphal cells in a previous study [28]. In several earlier reports, fengycin, iturin, and bacilomycin D were found to be associated with ROS accumulation and the subsequent death of fungal cells [56,57,58]. Moreover, the expression of ROS-scavenging genes was studied through RT-qPCR. We hypothesize that the possible mode of action adopted by fengycin in the suppression of *S. sclerotiorum* and one of the possible mechanisms could be the fengycin-induced accumulation of ROS in *S. sclerotiorum* hyphae, as revealed by the lower expression of ROS-scavenging genes in the pathogen. The expression of the three ROS-scavenging genes *SsSOD*, Ss*CAT1*, and *SsPO* was significantly downregulated upon treatment with fengycin. Like our study, the expression of ROS-scavenging genes *SOD* and *CAT* was reported to be dramatically reduced in *Magnaporthe grisea* hyphae as a response to treatment with fengycin [28]. In another instance, the expression of five putative ROS-scavenging genes, three catalases and two peroxidases, was highly downregulated in *Fusarium graminearum* hyphae after exposure to bacilomycin D produced by *B. amyloliquefaciens* FZB42 [58]. The downregulation of genes encoding ROS-scavenging enzymes weakens the pathogen’s ability to neutralize the effect of ROS and prevents cell death. Collectively, the induction of oxidative burst due to fengycin is associated with ROS accumulation and the subsequent death of fungal cells in our study. 

An in planta experiment revealed that the lesion size in fengycin-treated tomato plants was much reduced compared to infected control plants. In addition, the results of the transcriptional regulation analysis suggested that fengycin can upregulate the expression of plant defense-related genes involved in ISR to protect plants against *S. sclerotiorum* infection. The expression of six defense-related genes, including *SOD*, *PPO*, *PAL*, *GST*, *HMGR*, and *MPK3*, was significantly upregulated in fengycin-treated tomato plants. The results of our study demonstrated that fengycin was perceived by the plants as a defense elicitor and induced ISR in tomato plants against *S. sclerotiorum* by upregulating the expression of genes involved in defense-related pathways. Similar to our results, surfactin and fengycin have been previously reported to induce ISR in tomato and bean plants [23]. Several other reports have presented the role of lipopeptides in inducing ISR in plants [24,25]. We have observed a higher expression of these genes at 48 hpi in fengycin + *S. sclerotiorum* treatment compared to plants treated with fengycin only. This is because the plant pathogens release defense elicitors that trigger the induction of ISR in plants. As a result of host–pathogen interaction, the expression of defense-related genes is upregulated in the plants to resist the attack of the pathogen [59]. Therefore, defense elicitation by inoculating fengycin in pathogen-challenged plants resulted in a higher defense response in tomato plants than stand-alone treatments. The induction of ISR in plants is characterized by a series of complex spatio-temporal networks of metabolic activities. As a result of ISR elicitation, the molecular mechanisms involved in the rapid production of ROS and defense-related enzymes are triggered [60,61]. ROS react with lipids, proteins, and DNA, altering membrane integrity and causing cellular damage [62]. To overcome the damage due to ROS, plants produce several enzymatic and non-enzymatic ROS scavengers [21,62]. Previous reports have indicated that the elicitation of ISR was associated with the activation of defense-related enzymes [63,64]. 

The first line of defense in plants is the production of enzymatic antioxidants that can neutralize oxidative burst caused by superoxide anion (O_2_^−^) and hydrogen peroxide (H_2_O_2_). *SOD* is an enzymatic antioxidant that is essential to preventing the damage due to oxidative burst against all aerobic microorganisms [65]. The expression of *SOD*-encoding genes was significantly upregulated in our study. It has been reported that *SOD* activity increases under various biotic and abiotic stresses, conferring enhanced tolerance against the damage [66]. *SOD* catalyzes the conversion of O_2_^−^ to O_2_ and H_2_O_2_, which explains its vital role in plant defense. Enhanced production of H_2_O_2_ causes an upsurge in *SOD* activity [21]. The balance between *SOD* and H_2_O_2_-scavenging enzymes is crucial to determining the steady-state level of H_2_O_2_ and O_2_^−^ in the cells and preventing the damage [65]. *PPO* catalyzes the oxidation of phenols to toxic quinones and enhances the plant defense [19,21]. The overexpression of *PPO* in tomato plants has been reported to improve disease resistance, which is consistent with our result [19]. *PAL* is a key enzyme involved in phenylpropanoid metabolism leading to the production of defense-related antimicrobial compounds such as lignins, flavonoids, phytoalexins, and coumarins, in order to overcome the damage due to a pathogen attack [20]. In several previous reports, the overexpression of *PPO*, *POD,* and *PAL* was associated with ISR induction and enhanced disease suppression [67,68,69]. In agreement with our findings, surfactin produced by *Bacillus subtilis* BMG02 enhanced the expression of the *PAL* gene and induced ISR in tomato plants against tomato mosaic virus (ToMV) [27]. Similarly, surfactin and fengycin protected grapes from downy mildew by the stimulation of plant defense and upregulation of *PAL* and several other defense-related genes [70].

The over-overexpression of *MPK3*, *GST*, and *HMGR* genes in our study indicated their possible role in the ISR response. These genes and their subsequent products play different roles in plant defense. In early defense signaling mitogen-activated protein kinases (MPK), a cascade of signaling molecules hold a vital role in signal transduction to prepare the plants against the pathogen invasion [71,72]. *MPK3* is an important regulator of benzothiadiazole-induced elicitation of the defense response [73]. The overexpression of the *MPK3* gene has been reported to provide basal resistance against *Botrytis cinerea* and acts as an elicitor to trigger a defense response [73]. Glutathione S-transferase (*GST*) is a detoxifying enzyme involved in stress modulation pathways in plants [74]. *GST* plays catalytic and regulatory functions, providing physiological flexibility and enhanced tolerance against multiple biotic and abiotic stresses [75]. The over-expression of a rice *GST* gene was associated with enhanced tolerance in *Arabidopsis* against salinity and oxidative stresses [76]. The enzyme HMG-CoA reductase (3-hydroxy-3-methyl-glutaryl-coenzyme A reductase abbreviated as *HMGR*) catalyzes the first step in the mevalonate (MVA) pathway for isoprenoid biosynthesis that is modulated as a result of a pathogen attack [77,78]. Isoprenoids are significant for their role in plant defense against biotic and abiotic stresses. Like our results, previously, surfactin induced the overexpression of *GST* and *HMGR* genes and contributed to the activation of plant defense against *Zymoseptoria tritici* in wheat [79]. In support of our hypothesis, the expression of all these defense-related genes was up-regulated in the present study as a response to fengycin treatment to protect the plants against *S. sclerotiorum* infection. It can be concluded that fengycin plays an important role in plant defense elicitation against *S. sclerotiorum*.

## 5. Conclusions

Our study revealed the possible mechanism of action of fengycin and elucidated the specific role of fengycin in the elicitation of ISR in tomato plants against *S. sclerotiorum*. Fengycin efficiently induced ultrastructural changes and ROS accumulation in *S. sclerotiorum* and elicited the defense response in tomato plants against the pathogen by upregulating the expression of defense-related genes in plants. It strengthens the fact that fengycin possesses strong antifungal activity with multiple modes of action to suppress *S. sclerotiorum*. Further research is required to explore the potential of fengycin produced by *B. amyloliquefaciens* FZB42 over time. Fengycin should be evaluated at a field level and studies on biopesticide formulations need to be conducted to ascertain the biocontrol potential of fengycin produced by *B. amyloliquefaciens* FZB42 for the large-scale and long-term control of *S. sclerotiorum*.

## Figures and Tables

**Table 1:** List of oligonucleotides used to study the expression of ROS-scavenging genes in *Sclerotinia sclerotiorum* through RT-qPCR.

No.	Gene	Code	Primer
1	Peroxidase	*SsPO*-F	TGCTTGGCTTTGACTGGTTC
		*SsPO*-R	GCACTCTGCTGGAACATACG
2	Superoxidase distmutase	*SsSOD1*-F	GAGGTGACTCCAAGGTCTCC
		*SsSOD1*-R	GCAGATGTGCAACCGTTAGT
3	Catalase	*SsCat1*-F	GCTTCTGGTGCTTTCCACAA
		*SsCat1*-R	AACCGACAAGCTCAGTACCA
4	Actin	*SsActin*-F	CAACGATTGAGCGAGGATACA
		*SsActin*-R	CGACAAGATGAGGTTGGAAGAG

**Table 2:** List of primers used to study the transcriptional regulation of defense-related genes in tomato.

No.	Name	Code	Primer
1	Mitogen-activated protein kinase 3	*MPK3*-F	CAGGCAACTCCCACAACATC
		*MPK3*-R	CTAGTAGGGTCGAGCGTCAA
2	Polyphenol oxidase	*PPO*-F	CGCCTTACGTTCTTGGGAAT
		*PPO*-R	TTGAACCACGGACAGTACCA
3	3-hydroxy-3-methylglutaryl-coenzyme A reductase	*HMGR*-F	AGGGCCTTTGTTGCTTGATG
		*HMGR*-R	TGTCATGCCATCTCTGAGCA
4	Superoxide dismutase	*SOD*-F	GGGAGCAATACTACAGTTACTAAT
		*SOD*-R	GTTCTACTAATTTAAGCTTTGCCTT
5	Phenylalanine ammonia-lyase	*PAL*-F	AGCTGAGGCTGTTGACATCT
		*PAL*-R	CCTCCAAATGCCTCAAGTCG
6	Glutathione-S-transferase	*GST*-F	TCCTACACCTGATGTTGTCA
		*GST*-R	TCAAACACCTCTTAATTCAATTTGC
7	Actin	*ACTIN*-F	TGCCATTCTCCGTCTTGACT
		*ACTIN*-R	GCTGAGGTGGTGAACGAGTA
